# A trait database and updated checklist for European subterranean spiders

**DOI:** 10.1038/s41597-022-01316-3

**Published:** 2022-05-26

**Authors:** Stefano Mammola, Martina Pavlek, Bernhard A. Huber, Marco Isaia, Francesco Ballarin, Marco Tolve, Iva Čupić, Thomas Hesselberg, Enrico Lunghi, Samuel Mouron, Caio Graco-Roza, Pedro Cardoso

**Affiliations:** 1 LIBRe—Laboratory for Integrative Biodiversity Research, Finnish Museum of Natural History, University of Helsinki, Helsinki, Finland; 2 DarkMEG—Molecular Ecology Group, Water Research Institute, National Research Council of Italy (CNR), Verbania, Pallanza Italy; 3 Ruđer Bošković Institute, Zagreb, Croatia; 4 Croatian Biospeleological Society, Zagreb, Croatia; 5 Department of Evolutionary Biology, Ecology and Environmental Sciences, Biodiversity Research Institute (IRBio), Universitat de Barcelona, Barcelona, Spain; 6 Zoological Research Museum Alexander Koenig, Bonn, Germany; 7 Department of Life Sciences and Systems Biology, University of Turin, Torino, Italy; 8 Systematic Zoology Laboratory, Department of Biological Sciences, Tokyo Metropolitan University, Minami-Osawa, Hachioji-shi, Tokyo, Japan; 9 Department of Zoology, University of Oxford, Oxford, UK; 10 Key Laboratory of the Zoological Systematics and Evolution, Institute of Zoology, Chinese Academy of Sciences, Beijing, China; 11 Museo di Storia Naturale dell’Università degli Studi di Firenze, “La Specola”, Firenze, Italy; 12 Univ Lyon, Université Claude Bernard Lyon 1, CNRS, ENTPE, UMR 5023 LEHNA, F-69622 Villeurbanne, France; 13 Department of Geosciences and Geography, University of Helsinki, Helsinki, Finland

**Keywords:** Community ecology, Taxonomy, Conservation biology

## Abstract

Species traits are an essential currency in ecology, evolution, biogeography, and conservation biology. However, trait databases are unavailable for most organisms, especially those living in difficult-to-access habitats such as caves and other subterranean ecosystems. We compiled an expert-curated trait database for subterranean spiders in Europe using both literature data (including grey literature published in many different languages) and direct morphological measurements whenever specimens were available to us. We started by updating the checklist of European subterranean spiders, now including 512 species across 20 families, of which at least 192 have been found uniquely in subterranean habitats. For each of these species, we compiled 64 traits. The trait database encompasses morphological measures, including several traits related to subterranean adaptation, and ecological traits referring to habitat preference, dispersal, and feeding strategies. By making these data freely available, we open up opportunities for exploring different research questions, from the quantification of functional dimensions of subterranean adaptation to the study of spatial patterns in functional diversity across European caves.

## Abstract (summary)

**Table:** 

Measurement(s)	morphological trait • ecological trait
Technology Type(s)	literature extraction • observation
Sample Characteristic - Organism	Agelenidae • Amaurobiidae • Anapidae • Cybaeidae • Dysderidae • Hahniidae • Leptonetidae • Linyphiidae • Liocranidae • Mysmenidae • Nesticidae • Pholcidae • Pimoidae • Segestriidae • Sicariidae • Sparassidae • Symphytognathidae • Telemidae • Tetragnathidae • Theridiidae
Sample Characteristic - Environment	caves • subterranean habitats
Sample Characteristic - Location	Europe

## Background & Summary

Ecology is facing a ‘functional revolution’. Researchers are collecting species traits (glossary in Table 1) at unprecedented rates and depositing them in centralized databases focused on all kinds of organism including fungi^1,2^, plants^3,4^, and animals^5–11^. Traits are becoming an essential currency in ecology, especially to quantify functional diversity^12,13^. For example, traits allow us to bridge some of the conceptual gaps that exist between ecology and evolution^14^, to mechanistically explore ecological^15^ and biogeographical^16^ processes, as well as to prioritize species, habitats, and ecosystems for conservation^17–19^.

**Table 1:** Glossary of specialized term used.

Term (acronym)	Definition used in this paper
Functional diversity (FD)	Any measure of the diversity of traits of organisms composing a group, such as a community or an ecosystem^12^
Terrestrial subterranean habitat/ecosystem	All the subterranean spaces harbouring species showing traits typical to subterranean life. These include human-accessible natural subterranean spaces (i.e., caves), network of fissures with sizes smaller than the human scale, and artificial subterranean habitats (e.g., mines, blockhouses, cellars)^101^. Different spider species are able to occupy any or all of the habitats above^26^
Troglobiont/Troglophile	See section “*Ecological classification*”
Shallow Subterranean Habitat (SSH)	The subterranean habitats close to the surface, harbouring subterranean species, including epikarst, lava tubes, *Milieu Souterrain Superficiel* (MSS), deep leaf litter, and soil strata^102^
Trait	For the purpose of the paper, traits are intended in the broad sense of the World Spider Trait database, namely any phenotypic entity (e.g., morphological, anatomical, ecological, physiological, behavioural) measured at the species level^10,32^. In general, all traits are regarded as functional in that they are products of evolution and thus potentially linked to individual fitness^103^. However, almost always, the functional connotation of a trait is inferred based on indirect evidence

Despite all these promising developments, an impediment we are facing in advancing functional ecology is that there is still limited availability of trait databases for most organisms, especially those living in difficult-to-access, and hence generally less studied, ecosystems. Due to their inherent inaccessibility^20^ and several impediments to research^21^, caves and other subterranean habitats exemplify well this point. Whereas the community of subterranean biologists largely agrees that a functional perspective is key to better understand the ecology of caves^22^, trait-based subterranean studies remain unicorns^23–25^. The scarcity of centralized trait database for virtually any subterranean animal group means that we are still far from seeing the ‘dark side’ of the functional revolution as described above.

Spiders (Arachnida: Araneae) are a dominant component of subterranean biological communities, playing an important role as predators and showing various functional adaptations^26^. Despite their important role in subterranean food webs^27^, cave-dwelling spiders remain poorly known, especially in the tropics^28^. For most families of subterranean spiders, there is a general lack of distribution data, phylogenies, information of conservation status and, above all, traits. Europe is probably an exception to this general trend, given that there is a preliminary check list of subterranean spiders^29^, distributional and community composition data for selected caves and species scattered across the continent^30^, and growing information on their conservation status^31^.

**Fig. 1:**
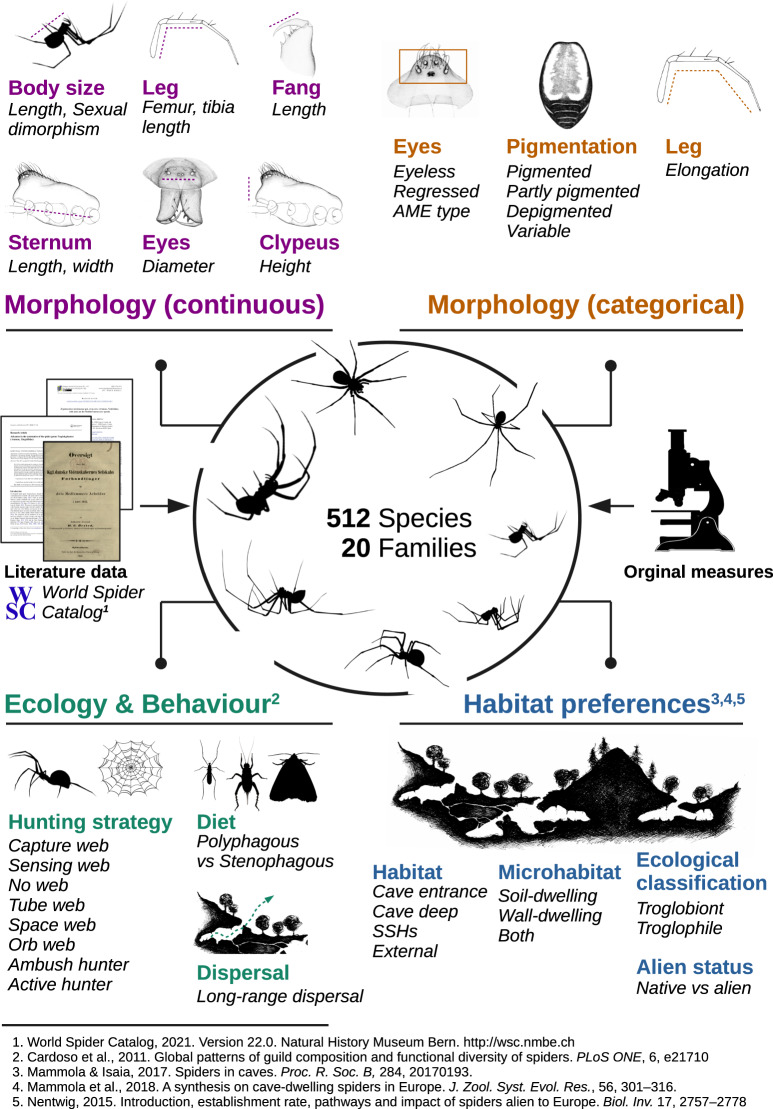
Infographic summarizing the study and the collected traits. See Table 2 for details about specific traits. All spider silhouettes are original drawings by Irene Frigo, except for the Pholcidae silhouette and the silhouettes of insects taken from *PhyloPics* (all with open licence). Original drawing of caves by Stefano Mammola.

Responding to the recent effort of the international arachnological community for compiling spider traits in an open centralized repository^10^, and building upon the launch of the first online version of the World Spider Trait database (WST)^32^, we present a functional trait database for all known species of European subterranean spiders. Integrating literature data and direct measures on specimens, we collected 64 morphological and ecological traits for all known subterranean spiders in Europe (Fig. 1). Here, we describe this trait database and make it freely available online. Finally, we discuss examples of trait-based research questions in subterranean biology hoping to promote the maximum use of this database.

## Methods

### Acronyms and jargon

A glossary of specialised terms used in this study is given in Table 1. The following acronyms are used: AME, Anterior Median Eyes; ALE, Anterior Lateral Eyes; PLE, Posterior Lateral Eyes; PME, Posterior Median Eyes; AME–ALE and PLE–PME, eyes distances; WST, World Spider Trait database. The notation NA (not available) is used for missing data in the database.

### Taxonomic and geographical coverage

We initially updated the checklist of European subterranean spiders provided in ref. ^29^ to obtain a list of species for which to collect relevant traits. Specifically, we:

I). included new species described after 2017;
II). included species that we had overlooked in the previous version of the checklist—see, e.g., missing species pointed out in ref. ^33^; and
III). updated taxonomy following recent nomenclatural changes^34^ and corrected a few mistakes we detected.

Concerning the geographical coverage of the checklist, we considered all European countries as defined in the Spiders of Europe^35^ database. However, we excluded North Africa, because it was only recently included in the Spiders of Europe, and oceanic islands (Azores, Madeira, and Canary Islands), because their insularity may lead to different processes shaping regional diversity^36^.

### Trait collection

For each species, we collected traits using literature data (mainly taxonomic descriptions) and, whenever specimens were available to us, direct measurements (27%; n = 137 species) (Fig. 1). We retrieved literature primarily from the World Spider Catalog^34^—i.e., the main repository of bibliography on the taxonomy of spiders^37^—and secondarily from the Spiders of Europe repository and Google Scholar. We used the latter two sources to retrieve most literature on ecological traits.

#### Morphological measures

We collected several traits that different authors have considered proxies for body size, food specialization, and subterranean adaptation^38–41^ (Table 2). All length measures are given in millimetres.

**Table 2:** List of traits collected in this study. See section “Acronym and jargon” for a definition of all acronyms used.

Type	Trait	Measures	Source	Explanation and/or functional meaning
Morphology (continuous)	Body size (and sexual size dimorphism)	Minimum, Maximum, Average for females and males. Sexual size dimorphism is further calculated as body size male / female.	Direct measure when fresh specimens were available to us. Alternatively, literature data (original description or re-descriptions). Literature used is reported in the column “Citation”.	In subterranean species, body size is possibly related to habitat (pore) size^104^. Difference in size between females and males may provide indirect information on sexual selection mechanisms operating in subterranean habitats.
	Leg (and Leg elongation)	Femur I and tibia I length for females and males, and the average of males and females. Femur and tibia elongation is further calculated by dividing the average length and body size.		Leg length is a *proxy* for overall body size^105^. In subterranean spiders, leg length is often related with habitat (pore) size^106^ and leg elongation preferentially occurs in subterranean species^107^.
	Prosoma (size and shape)	Prosoma length, width, and height for females and males.		A *proxy* for overall body size^108^. Shape may vary according to different microhabitats. In certain species, prosoma height is hypothesized to be a proxy measure of subterranean adaptation—i.e., flattening of the prosoma profile with increasing adaptation^39,107^.
	Cheliceral fang	Fang length for females and males.		The dimension of cheliceral fangs provides information on dietary requirements^104^.
	Clypeus	Clypeus height for females and males.		Same as prosoma height.
	Eyes	Diameter of AME, ALE, PME, and PLE. Distance AME–ALE and PME–PLE. Note that a variable “AME_type” describes whether AME are present or missing due to either subterranean adaptation or ontology (six-eyed families; see main text).		In spiders, eye regression is among the most evident morphological change to the subterranean conditions^26^. Regression of different groups of eyes provide indication for different degree of adaptation. For example, in *Troglohyphantes*, the anterior median eyes are usually the first undergoing regression^106^.
Morphology (Categorical)	Eyes	Binary variables (0 = no; 1 = yes) indicating whether the species has regressed eyes or is eyeless (non-functional eyes). Note that a species can both have regressed eyes and eyeless status when different population exhibit different degrees of eye regression.	Mainly literature data (original description or re-descriptions). Literature used is reported in the column “Citation”.	See “Eyes (and ratios)”.
	Pigmentation	Ordinal variables, indicating whether the species is pigmented, variable, partly pigmented, depigmented.		In spiders, with the adaptation to the subterranean conditions, body pigment is generally the first morphological character to get lost^26^.
	Leg elongation	Binary variables (0 = no; 1 = yes) indicating whether the species has elongated legs.		See “Leg (and Leg elongation)”.
Ecology & Behaviour	Guild	Categorical variable indicating the general functional guild of each spider: Ambush, Ground, Orb, Other, Sensing, Sheet, Space, Sheet-space, or Specialist. Note that the guild ‘Sheet-space’ is not originally coded in Cardoso *et al*.^102^. It has been introduced for Pholcidae based on the expert opinion of BH.	Based on literature data and/or our expert opinion.	A general summary of the hunting ecology of each species^43^.
	Hunting strategy	Binary variables (0 = no; 1 = yes) indicating the species web strategy (Capture web, Sensing web, and no web). For each species, we also indicated the type of web if any (Tube web, Sheet web, Space web, Orb Web) and/or the type of active hunting strategy if any (Ambush hunter or Active hunter).		Spiders are important predators in caves; different types of hunting strategies may be associated to different microhabitats. Furthermore, the subterranean environment selects for specific hunting strategies^43^.
	Diet (Food specialist)	Binary variables (0 = no; 1 = yes) indicating whether the species is a food specialist or not.		Food specialisation is thought to be rare in subterranean communities given the general scarcity of food^26^. However, food specialisation seems to be retained in a few species (e.g., Dysderidae^41^) and may be associated with niche differentiation to avoid direct competition^109^.
	Dispersal	Binary variables (0 = no; 1 = yes) indicating whether the species can perform long range dispersal outside the cave.		Long range dispersal is rare in subterranean species, and may be only found in generalist species with limited affinity to subterranean habitats.
Habitat preference	Ecological classification	Categorical variable indicating whether the species is a Troglobiont or a Troglophile (see section “*Ecological classification*” for a definition).	Based on literature data and/or our expert opinion.	Gives a rough indication of the level of dependency of each species to the subterranean medium. See section “*Ecological classification*” for some cautionary arguments.
	Alien status	Binary variable (0 = no; 1 = yes) indicating whether the species is considered an alien species in Europe or not (*sensu* ref. ^43^).		Subterranean habitats are thought to be poorly permeable to invasion by alien species^49^. Still a few alien elements have been documented, especially in disturbed habitats (e.g. mines)—see overview and discussion in ref. ^110^.
	Habitat	Binary variables (0 = no; 1 = yes) indicating whether the species occur in Deep caves, at Cave entrances, in SSHs, or in External habitats. Note that a single species can occur in multiple of these.		Gives a rough indication of the type of subterranean habitats occupied by each species. The ability of a species to occupy multiple habitats provide indication on its general plasticity.
	Verticality	Categorical variable indicating whether a species in a cave preferentially dwell on the ground, on the walls, or both. Note that a single species can occur in multiple of these.		In a typical subterranean community, different species are often adapted to different microhabitats. In spiders, for example, there can be a niche differentiation between wall and soil-dwelling species^111^.

For measured species, we used averaged values whenever multiple specimens were available to us. If total body length was not reported in the original description, we approximated it as the sum of prosoma + opisthosoma. Also, in a few descriptions, authors reported tibia length as the sum tibia + patella. In such cases, we approximated tibia length as a fraction of the value based on the ratio tibia/patella in congeneric species.

In six-eyed spider families (Dysderidae, Leptonetidae, Sicariidae, Symphytognathidae, and Telemidae), for which a pair of eyes is missing due to ontological reasons unrelated to subterranean adaptation, we assumed the missing pair of eyes to be AME^42^. If a paper reported the missing pair of eyes to be ALE, we ignored the information and assumed AME to be the missing one. We assigned a value of 0 to the missing pair of AME in six-eyed families. However, to distinguish these from missing AME due to cave adaptation in eight-eyed spider families, which were also scored as zeros, we included the ontology of eyes as a variable in the dataset (categorical variable ‘AME_type’ made up of three levels: “Present”, “Absent_Ontology”, and “Absent_Adaptation”). This variable can be included in some analyses to ensure that, in functional diversity calculations, the ontologically missing AME do not mix with missing AME due to subterranean adaptation.

#### Habitat and ecological traits

We classified ecological traits (Table 2) based on ref. ^43^. We included functional guild, foraging strategy (type of web and method of active hunting), and prey range (specialist or generalist). Conversely, we excluded vertical stratification (ground or vegetation) and circadian activity (diurnal or nocturnal), as these are not relevant for subterranean ecosystems. Instead, we classified vertical stratification in a cave (ground, wall, or both) and potential for long-range dispersal outside subterranean habitats (e.g., ballooning in *Meta* spiders^44^ or active dispersal on the ground in *Pimoa*^45^).

#### Ecological classification

In the previous version of the checklist of European subterranean spiders, we also reported an indication of the level of affinity of each species to the subterranean medium^46,47^. This was assessed by the group expert involved in this paper or taken from literature (whenever this information was mentioned in the original description), and included two categories: i) Troglophile, for species able to maintain stable subterranean populations or inclined to inhabit subterranean habitats, being, however, associated with surface habitats for some biological functions or able to maintain surface populations too; and ii) Troglobiont, for species strictly bound to subterranean habitats. For consistency, we included this ecological classification in this update of the checklist and in the trait database. However, since definition of ecological categories is traditionally a stumbling stone of biospeleology^48,49^, and sparkled some debate in the form of personal communications, we would like to clarify its real meaning. The attribution of some species to one category or another may be problematic as this is not a strictly categorical trait but often can be seen as a continuum from troglobionts to surface dwellers—including the intermediate troglophiles. As we see it, this is just a practical tool that allows one to roughly subdivide groups of species in broad macro-categories. The proper way for assessing the species affinity for subterranean or surface habitats would be a systemic survey including extensive sampling primarily in the surface habitats, population studies, and a robust phylogenetic framework^47^, all of which are practically non-existent for most subterranean spiders. There are, however, alternative ways to do so depending on the research questions of interest^50^. For example, one can by-pass this classification and simply use morphological traits such as eye regression, leg elongation, and pigmentation as a *proxy* for the subterranean specialization of each species.

### Data visualisation

To illustrate the usage of the dataset, we plotted the distribution of key traits as density plot with the R library ‘ggplot2’^51^. We also generated a representation of the trait space for European subterranean spiders showing its general organisation and the position of each spider family within it. To this end, we selected a subset of traits from the whole trait matrix, representing:

I). General morphology of species (Average body size, Sexual size dimorphism, and Prosoma shape);
II). Morphological adaptation to subterranean condition, including both categorical (Pigmentation, Presence/absence of Eyes, Eye regression, Leg elongation, AME, ALE, PME, and PLE, and AME type) and continuous (Femur elongation and Profile reduction) traits;
III). Hunting strategy (all binary variables referring to hunting strategy and diet, as well as the continuous variable Fang length);
IV). Dispersal behaviour (Dispersal); and
V). Microhabitat occupation (Verticality).

We performed data exploration following recommendations in ref. ^52^, checking variable distribution, multicollinearity among continuous traits via Pearson’s *r* correlations, and presence of missing data. As a result of data exploration, we excluded Fang length and Profile reduction because they contained more than 80% of missing values. To homogenize variable distribution, we log-transformed all continuous variables that do not assume negative values. We also standardized all continuous traits to mean = 0 standard deviation = 1 to ensure comparable ranges among traits.

Since the trait matrix contains both continuous, binary, and categorical variables, we used a Gower distance to estimate trait dissimilarity among species^53^. Because different traits span different functional roles, we used an optimisation method to attribute weight to traits within groups^54^. To this end, we assigned traits to the five groups of variables as defined above.

We visualized the trait space as the first two axes of a principal coordinate analysis using the trait dissimilarity matrix as input data, using the R package ‘ape’ version 5.5.0^55^. For graphical visualisation, we estimated density of species onto the ordination diagram using a kernel density. Furthermore, we visualized the centroid of each family to get an overview of the spatial relationships among families within the trait space. To relate traits to ordination axes, we used the function *envfit* from the R package ‘vegan’ version 2.5.7^56^. This function calculates a multiple linear regression of the traits (dependent variable) and species scores on ordination axes (independent variables). The normalized regression coefficients multiplied by the square root of the coefficient of determination are used to position the trait onto the ordination diagram. Note that this analysis was only possible for complete cases—i.e., species without missing traits (N = 154). We performed all analyses in R version 4.1.0^57^.

## Data Records

The trait database is available in Figshare^58^ as a tab-delimited file (.csv) and in Excel (.xlsx) format. Traits missing from the World Spider Trait database were also deposited therein—accessible directly in R environment using the function *traits* in the R package ‘arakno’^59^.

**Fig. 2:**
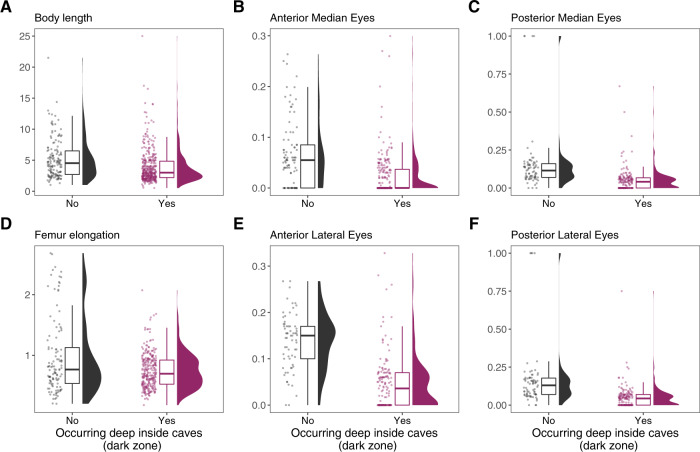
Variation in selected traits values of subterranean spiders split by whether a species occurs in deep caves (obscure zone) or not. Jittered points are the actual values, boxplots summarize median and quantiles, and density plots summarize data distribution. All length measures are in millimetres. (**A**) Average Body length (male and female are averaged). (**B**) Average size of the Anterior Median Eyes. (**C**) Average size of the Posterior Median Eyes. (**D**) Femur elongation, calculated as the ratio between the Femur I length and the average Body length. (**E**) Average size of the Anterior Later Eyes. (**F**) Average size of the Anterior Lateral Eyes.

**Fig. 3:**
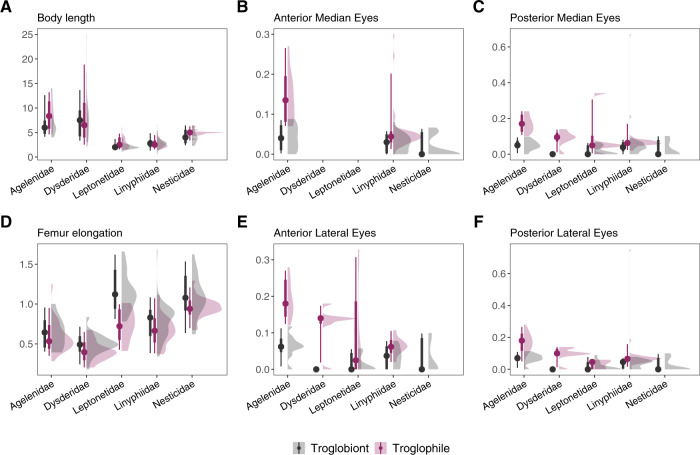
Variation in selected traits values of dominant families of spiders in European caves split by Ecological classification. Density plots summarize data distribution, dots are median values, and bars summarize quantiles. All length measures are in millimetres. (**A**) Average Body length. (**B**) Average size of the Anterior Median Eyes; no data are shown for Leptonetidae and Dysderidae, both of which are six-eyed spider families. (**C**) Average size of the Posterior Median Eyes. (**D**) Femur elongation, calculated as the ratio between the Femur I length and the average Body length. (**E**) Average size of the Anterior Later Eyes. (**F**) Average size of the Anterior Lateral Eyes.

Detailed explanation of traits, including their hypothesized functional meaning, is given in Table 2. The dataset consists of 64 traits (some examples of trait distributions are given in Fig. 2) for 520 species belonging to 20 families associated with caves (Table 3)—34 species more than in the previous checklist^29^. The family comprising most species is Linyphiidae (224 species, almost half of them belonging to a single genus *Troglohyphantes*), followed by Dysderidae (62 species), Leptonetidae (60), Nesticidae (56), and Agelenidae (43). All these families comprise several specialized species only found in subterranean habitats and showing traits such as full eye regression and complete depigmentation, but also generalist species exhibiting a low degree of morphological specialisation to subterranean life (Fig. 3). The remaining families are all represented by up to 30 species and encompass spiders with diverse levels of subterranean specialisation. We refer the reader to ref. ^29^. for an in-depth taxonomic and biogeographical account.

**Table 3:** Spider families with number of genera and species occurring in subterranean habitats across Europe—updated from ref. ^29^. Total number of genera for each family are derived from Spiders of Europe^35^.

Family	Subterranean species		Total in Europe		Notes about European subterranean species
	Gen	Sp	Gen	Sp	
Agelenidae	5	43	22	237	Several species associated with caves. Eyeless species in *Histopona* and *Hadites*.
Amaurobiidae	1	3	4	43	Black lace-weaver spiders (*Amaurobius*) often occur at cave entrances^112^.
Anapidae	2	3	3	5	Some species are found in SSHs.
Cybaeidae	1	1	7	27	*Cybaeus vignai* is the only species associated with subterranean habitats^113^.
Dysderidae	15	62	23	426	Many subterranean species, with various degrees of subterranean specialization, mostly distributed in the Mediterranean basin.
Hahniidae	5	8	7	31	Some cave-dwelling species.
Leptonetidae	8	60	8	72	Many species and genera related the subterranean habitats with different levels of subterranean specialization^114^.
Linyphiidae	30	224	222	1368	Different genera related to subterranean habitats. Among these, *Troglohyphantes* is the genus with most subterranean species in Europe^115^.
Liocranidae	2	5	12	66	Specialized subterranean species in *Cybaeodes* and *Agraecina*.
Mysmenidae	1	1	3	4	*Trogloneta granulum* in deep strata of screes and other SSHs^116^.
Nesticidae	8	56	8	57	Many species and genera related the subterranean habitats with different levels of subterranean specialization.
Pholcidae	7	28	15	63	Many species associated with caves, such as in genera *Hoplopholcus*^117^ and *Stygopholcus*^118^, none of which is fully specialized to cave life^40^.
Pimoidae	1	4	1	4	Represented in Europe by four species of *Pimoa*^45^.
Segestriidae	1	2	2	18	Two species of *Segestria* found in caves.
Sicariidae	1	1	1	4	*Loxosceles rufescens* is often associated with caves and other SSHs.
Sparassidae	1	1	7	40	*Heteropoda variegata* is often associated with caves.
Symphytognathidae	1	1	1	1	Only represented in Europe by *Anapistula ataecina*^119^.
Telemidae	1	1	1	1	Only represented in Europe by *Telema tenella*^120^.
Tetragnathidae	2	3	6	38	Representatives of *Meta* and *Metellina* are widespread in European caves, where they typically occur in the entrance area^121^.
Theridiidae	3	5	48	262	Some subterranean species in *Episinus*, *Robertus* and *Rugathodes*.
Total (20)	96	512	720	5326	

SSH: Shallow Subterranean Habitat (see Glossary in Table 1); Gen: Number of genera; Sp: Number of species.

## Technical Validation

**Fig. 4:**
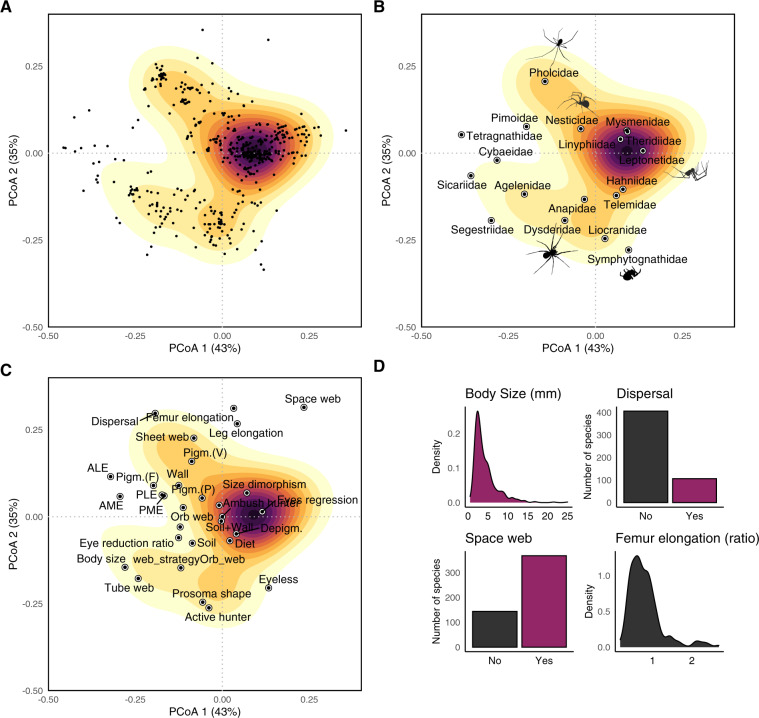
Example of a trait space representation for European subterranean spiders. (**A**) Distribution of European subterranean spiders along the first two axes of a principal coordinate analysis describing the trait similarity among species. Gradient of colour denote density of species—higher density in darker areas. (**B**) Same as the representation of the trait space in **A**, but mapping the centroid of each family onto the ordination diagram. (**C**) Same as the representation of the trait space in **A**, but mapping the position the trait onto the ordination diagram. (**D**) Distribution of values of the four traits that contribute the most to determining the distribution of species in the trait space (untransformed values are shown). Original spider silhouettes by Irene Frigo, except for the Pholcidae silhouette by Sean McCann via *PhyloPics* (Public Domain Dedication 1.0 license) and Symphytognathidae by CG-R.
III). Given the scale of the dataset and the lack of multiple specimens for most species considered, this dataset do not contain information on intraspecific variability—one exception being the minimum-maximum range for body size. It is well known that intraspecific trait variability is an important aspect of community ecology^64,65^, which can be pronounced in many taxa^66,67^. This is seemingly true also for subterranean spiders. For example, in the well-studied case of Western Alpine *Troglohyphantes* (Linyphiidae), intraspecific variability has been reported for morphological traits relating to subterranean adaptation^38,39,68^, but also in individual thermal tolerance^68^. Likewise, individuals of *Kryptonesticus eremita* (Simon, 1880) (Nesticidae) may show different levels of pigmentation depending on how far from the cave entrance they are collected^69^. Accordingly, any analysis based on this database must be taken as an average representation of the process under study, and the information relativized accordingly^70^.

There are some limitations that one must be aware of when using the dataset:

I). Given the low availability of specimens—many of these species have been collected at the time of their description and never recorded thereafter—the dataset is a mixture of literature data and direct measures. It includes families for which we have been able to measure all species (Pholcidae) and others for which over half of species traits are derived from original description and other literature sources (e.g., Linyphiidae and Leptonetidae). Unfortunately, many original descriptions, both recent and old, contain poor information, limiting the possibility to extract traits.
II). For the same reason, there is a high frequency of missing data for some traits and species. This means that one may want to focus on traits that are well sampled and use a reduced matrix of only well-sampled traits in community-level analyses. There are statistical ways to partly remedy these problems. Different imputation methods can be used to infer missing trait values^60,61^. Most of these imputation tools are implemented in the function *fill* in the R package ‘BAT’^62,63^. Also, in community-level analyses, one can use functional distance measures able to accommodate missing data, especially Gower distances^54^. The latter method is the one we used to generate the trait space in Fig. 4.

## Usage Notes

### Complementarity with other databases

The database is fully compatible with community composition data for European cave spiders available in Figshare^71^ and cave spider occurrence records deposited in the Global Biodiversity Information Facility (GBIF)^30^. Traits can be univocally linked to each species included in the latter datasets (or others) using the species Latin name. However, one may need to check and update the taxonomy of some species depending on the latest nomenclature changes. This can be done automatically using the function *checknames* in the R package ‘arakno’^59^, which checks for nomenclature changes, synonyms, and spelling errors dialoguing with the most up-to-date version of the World Spider Catalog^34^.

### Example of trait-based research questions in subterranean biology

As old as the recognition of the bizarre morphology of cave species is the search for ecological and evolutionary explanations for these unique adaptations^72^. Trait-based ecology is a critical framework to this end^73^. By focusing on how traits interact mechanistically with environments across spatial scales and levels of organization, we can use geographically ubiquitous and ecologically diverse cave spiders to test hypotheses in subterranean biology and beyond^22^. Here, we provide some examples of avenues of research, hoping to both stimulate re-use of the dataset and the quest for developing similar databases for both spiders outside Europe and for other subterranean taxa.

#### Quantifying functional redundancy and subterranean specialization

Different spider species and families occupy distinct regions of the trait space (Fig. 4). The position of the species in the trait space can be mapped to obtain a quantification of their functional redundancy (e.g., if multiple species fall within highly sampled areas of the trait space). Within a given group (e.g., family or genus), one could also rank species according to their degree of adaptation by using traits relating to subterranean adaptation and calculating the functional distance of each troglobiont species from the average troglophile species or the closest surface species^50^—following the saying “*nothing [makes] sense in speleobiology without a comparison of cave animals with the ‘normal’ epigean ones*”^74^. Ultimately, the quantification of the level of subterranean specialization of species on a continuous scale allows us to explore the degree to which the specialization of a given community relates to local environmental conditions, interspecific interactions, and more.

#### Trait-based (macro)ecology of subterranean spiders

Traits are a useful aid for answering a range of questions in community ecology and macroecology^12,15,16,75^. To what extent is there functional convergence in the functional space of subterranean spider communities in a given region? Do different microhabitats within a cave select for functionally unique spiders? What is the maximum degree of functional similarity that allows two or more species to occupy the same environment? How does the functional space of a given subterranean community change after a perturbation event (e.g., the extinction of some species, the invasion by a non-native species)?

Similar questions can be answered using metrics such as community weighted trait means^76^ or more advanced ways to calculate the functional richness, dispersion, and regularity of the trait space occupied by a given community (e.g., functional dendrograms^77^ or probabilistic hypervolumes^78^). We refer the reader to recent accounts on functional diversity analyses for operation details about similar analyses^12,13^. Also, all these questions can be explored at different scales, from local communities inhabiting a single cave or cave system up to entire karst areas and even continents. This latter possibility is enhanced by the availability of broad-scale distribution and community composition data for European cave spiders^30^. For example, a recent study demonstrated that there is a quick turnover in the taxonomic diversity of subterranean spiders across Europe, mediated primarily by the geographic distance among caves and secondarily by the climatic conditions and availability of karst habitat, ensuring cave connectivity^79^. The usage of traits enables us to test whether the same distance decay occurs with respect to functional diversity, or if taxonomically distinct communities in caves can fulfil similar functional roles thereby determining a lower turnover in functional diversity in Europe. Whereas it is well-known that taxonomic and functional diversity decays at different rates along geographical and environmental gradients across different terrestrial and marine habitats and organisms^80^, similar patterns have never been explored in subterranean habitats.

#### Trait-based conservation of subterranean spiders

Species traits can be useful in conservation science, for example to assess species extinction risk, to prioritize species and habitat for conservation, and ultimately to define long-term conservation strategies^81^.

At the individual level, there has been recent interest in understanding the relationship between species traits and extinction risk, namely whether species possessing specific traits (e.g. larger body size, greater longevity) are more prone to extinction^82,83^. To the best of our knowledge, similar considerations have never been applied to subterranean species, let alone spiders.

At the community level, one can identify species with unique and original traits (‘functional outliers’ *sensu* ref. ^84^) versus species falling within densely populated regions of the trait space. This enables the possibility to map the extinction risk across a given global functional spectrum (e.g., the functional space of European cave spiders in Fig. 4) and ultimately to provide general guidance of where to focus in the search for priority species for conservation^85^. The rationale behind this possibility is that functionally unique species are often irreplaceable, whereas the ecological role of functionally redundant species can be performed by functionally analogous species in the community (‘biological insurance’ *sensu* ref. ^86^).

Historically, subterranean ecosystems have been overlooked in global biodiversity conservation agendas^87^. In recent years, as the conservation importance of subterranean ecosystems is being reaffirmed, there is a growing need to develop objective ways to prioritize subterranean species and regions to protect. There are several examples of studies proposing operational indexes targeting top-priority caves or subterranean sites for protection given a scenario of limited resources invested in conservation^88–90^. These high-priority sites usually end up corresponding with so-called “hotspots of subterranean diversity^91^”. However, in our view, all these prioritization attempts fail short on one key aspect: they only consider number of species and/or endemism in their protocol to design protected areas or conservation priorities—but see, e.g., ref. ^92,93^. A modern take on this subject would be to not only consider taxonomic diversity and relative measures, but also to maximize phylogenetic and functional diversity within a given protected area^94,95^, and even the extent to which species niches are accounted for^96^. A trait dataset such as the one released in this work is a first, necessary step towards the goal of obtaining a multi-pronged prioritization that accounts for multiple biodiversity facets^97^. This is of the utmost importance given the current threats on subterranean ecosystems, and the unique conservation challenges associated with these biota^98–100^.

## Data Availability

Annotated markdown R code used to generate the analysis is available in GitHub (https://github.com/StefanoMammola/European-cave-spider-traits-1.git).

## References

[1] Zanne AE (2020). Fungal functional ecology: bringing a trait-based approach to plant-associated fungi. Biol. Rev..

[2] Põlme S (2020). FungalTraits: a user-friendly traits database of fungi and fungus-like stramenopiles. Fungal Divers..

[3] Fraser LH (2020). TRY—A plant trait database of databases. Glob. Chang. Biol..

[4] Kattge J (2020). TRY plant trait database - enhanced coverage and open access. Glob. Chang. Biol..

[5] Oliveira BF, São-Pedro VA, Santos-Barrera G, Penone C, Costa GC (2017). AmphiBIO, a global database for amphibian ecological traits. Sci. Data.

[6] Lecocq T (2019). TOFF, a database of traits of fish to promote advances in fish aquaculture. Sci. Data.

[7] Jones KE (2009). PanTHERIA: a species-level database of life history, ecology, and geography of extant and recently extinct mammals. Ecology.

[8] Parr CL (2017). GlobalAnts: a new database on the geography of ant traits (Hymenoptera: Formicidae). Insect Conserv. Divers..

[9] Homburg K, Homburg N, Schäfer F, Schuldt A, Assmann T (2014). Carabids.org – a dynamic online database of ground beetle species traits (Coleoptera, Carabidae). Insect Conserv. Divers..

[10] Lowe, E. C. *et al*. Towards establishment of a centralized spider traits database. *J. Arachnol*. **48** (2020).

[11] Tobias JA (2022). AVONET: morphological, ecological and geographical data for all birds. Ecol. Lett..

[12] Mammola S, Carmona CP, Guillerme T, Cardoso P (2021). Concepts and applications in functional diversity. Funct. Ecol..

[13] de Bello, F. *et al*. *Handbook of trait-based ecology: from theory to R tools*. (Cambridge University Press, 2021).

[14] Edwards KF (2018). Evolutionarily stable communities: a framework for understanding the role of trait evolution in the maintenance of diversity. Ecol. Lett..

[15] McGill BJ, Enquist BJ, Weiher E, Westoby M (2006). Rebuilding community ecology from functional traits. Trends Ecol. Evol..

[16] Violle C, Reich PB, Pacala SW, Enquist BJ, Kattge J (2014). The emergence and promise of functional biogeography. Proc. Natl. Acad. Sci..

[17] Kosman E, Burgio KR, Presley SJ, Willig MR, Scheiner SM (2019). Conservation prioritization based on trait‐based metrics illustrated with global parrot distributions. Divers. Distrib..

[18] Cadotte MW, Carscadden K, Mirotchnick N (2011). Beyond species: functional diversity and the maintenance of ecological processes and services. J. Appl. Ecol..

[19] de Bello F (2010). Towards an assessment of multiple ecosystem processes and services via functional traits. Biodivers. Conserv..

[20] Ficetola GF, Canedoli C, Stoch F (2019). The Racovitzan impediment and the hidden biodiversity of unexplored environments. Conserv. Biol..

[21] Mammola S (2021). Collecting eco-evolutionary data in the dark: Impediments to subterranean research and how to overcome them. Ecol. Evol..

[22] Mammola S (2020). Fundamental research questions in subterranean biology. Biol. Rev..

[23] Cardoso P (2012). Diversity and community assembly patterns of epigean vs. troglobiont spiders in the Iberian Peninsula. Int. J. Speleol..

[24] Fernandes CS, Batalha MA, Bichuette ME (2016). Does the cave environment reduce functional diversity?. PLoS One.

[25] Saccò M (2019). New light in the dark - a proposed multidisciplinary framework for studying functional ecology of groundwater fauna. Sci. Total Environ..

[26] Mammola S, Isaia M (2017). Spiders in caves. Proceedings of the Royal Society B: Biological Sciences.

[27] Parimuchová A (2021). The food web in a subterranean ecosystem is driven by intraguild predation. Sci. Rep..

[28] Bloom T (2014). Discovery of two new species of eyeless spiders within a single Hispaniola cave. J. Arachnol..

[29] Mammola S, Cardoso P, Ribera C, Pavlek M, Isaia M (2018). A synthesis on cave-dwelling spiders in Europe. J. Zool. Syst. Evol. Res..

[30] Mammola S (2019). Continental data on cave-dwelling spider communities across Europe (Arachnida: Araneae). Biodivers. Data J..

[31] Milano F (2021). Spider conservation in Europe: a review. Biol. Conserv..

[32] Pekár S (2021). The World Spider Trait database (WST): a centralised global open repository for curated data on spider traits. Database.

[33] Ledesma E, Jiménez-Valverde A, de Castro A, Aguado-Aranda P, Ortuño VM (2019). The study of hidden habitats sheds light on poorly known taxa: spiders of the Mesovoid Shallow Substratum. Zookeys.

[34] World Spider Catalog. World Spider Catalog. Version 23.0. *Natural History Museum Bern* 10.24436/2 (2022).

[35] Nentwig, W. *et al*. Araneae - Spider of Europe. 10.24436/1 (2021).

[36] Malumbres-Olarte J (2021). Habitat filtering and inferred dispersal ability condition across-scale species turnover and rarity in Macaronesian island spider assemblages. J. Biogeogr..

[37] Nentwig W, Gloor D, Kropf C (2015). Spider taxonomists catch data on web. Nature.

[38] Mammola S (2020). Environmental filtering and convergent evolution determine the ecological specialization of subterranean spiders. Funct. Ecol..

[39] Mammola S (2018). Ecological speciation in darkness? Spatial niche partitioning in sibling subterranean spiders (Araneae: Linyphiidae: *Troglohyphantes*). Invertebr. Syst..

[40] Huber BA (2018). Cave-dwelling pholcid spiders (Araneae, Pholcidae): A review. Subterr. Biol..

[41] Arnedo MA, Oromí P, Múrria C, Macías-Hernández N, Ribera C (2007). The dark side of an island radiation: systematics and evolution of troglobitic spiders of the genus *Dysdera* Latreille (Araneae:Dysderidae) in the Canary Islands. Invertebr. Syst..

[42] Ubick, D., Paquin, P., Cushing, P. E. & Duperre, N. *Spiders of North America: An Identification Manual*. (Amer Arachnological Society, 2007).

[43] Cardoso P, Pekár S, Jocqué R, Coddington JA (2011). Global patterns of guild composition and functional diversity of spiders. PLoS One.

[44] Smithers P (2005). The early life history and dispersal of the cave spider *Meta menardi* (Latreille, 1804) (Araneae: Tetragnathidae). Bull. Br. arachnol. Soc.

[45] Mammola S, Hormiga G, Arnedo MA, Isaia M (2016). Unexpected diversity in the relictual European spiders of the genus *Pimoa* (Araneae:Pimoidae). Invertebr. Syst..

[46] Sket B (2008). Can we agree on an ecological classification of subterranean animals?. J. Nat. Hist..

[47] Trajano E, de Carvalho MR (2017). Towards a biologically meaningful classification of subterranean organisms: A critical analysis of the schiner-racovitza system from a historical perspective, difficulties of its application and implications for conservation. Subterr. Biol..

[48] Martínez A, Mammola S (2021). Specialized terminology reduces the number of citations to scientific papers. Proc. R. Soc. B Biol. Sci..

[49] Mammola S (2019). Finding answers in the dark: caves as models in ecology fifty years after Poulson and White. Ecography.

[50] Mammola, S. *et al*. Quantifying troglomorphism in hyperspace. *Arpha Conf. Abstr*. **5**, e82941 (2022).

[51] Wickham, H. *ggplot2: Elegant Graphics for Data Analysis*. (Springer-Verlag, 2016).

[52] Palacio FX (2022). A protocol for reproducible functional diversity analyses. EcoEvoRxiv.

[53] Gower JC (1971). A General Coefficient of Similarity and Some of Its Properties. Biometrics.

[54] de Bello F, Botta-Dukát Z, Lepš J, Fibich P (2021). Towards a more balanced combination of multiple traits when computing functional differences between species. Methods Ecol. Evol..

[55] Paradis E, Schliep K (2019). Ape 5.0: An environment for modern phylogenetics and evolutionary analyses in R. Bioinformatics.

[56] Oksanen, J. *et al*. R Package vegan: community ecology package. R package version 2.5-3 (2018).

[57] R Core Team. R: A language and environment for statistical computing. (2021).

[58] Mammola S (2022). Figshare.

[59] Cardoso P, Pekar S (2022). arakno – An R package for effective spider nomenclature, distribution, and trait data retrieval from online resources. J. Arachnol..

[60] Johnson TF, Isaac NJB, Paviolo A, González-Suárez M (2021). Handling missing values in trait data. Glob. Ecol. Biogeogr..

[61] Podani J, Kalapos T, Barta B, Schmera D (2021). Principal component analysis of incomplete data – A simple solution to an old problem. Ecol. Inform..

[62] Cardoso, P., Mammola, S., Rigal, F. & Carvalho, J. C. BAT: Biodiversity Assessment Tools. R package version 2.6.0 (2021).

[63] Cardoso P, Rigal F, Carvalho JC (2015). BAT – Biodiversity Assessment Tools, an R package for the measurement and estimation of alpha and beta taxon, phylogenetic and functional diversity. Methods Ecol. Evol..

[64] De Bello F (2011). Quantifying the relevance of intraspecific trait variability for functional diversity. Methods Ecol. Evol..

[65] Violle C (2012). The return of the variance: intraspecific variability in community ecology. Trends Ecol. Evol..

[66] Gentile G, Bonelli S, Riva F (2020). Evaluating intraspecific variation in insect trait analysis. Ecol. Entomol..

[67] Wong MKL, Carmona CP (2021). Including intraspecific trait variability to avoid distortion of functional diversity and ecological inference: Lessons from natural assemblages. Methods Ecol. Evol..

[68] Mammola S, Piano E, Malard F, Vernon P, Isaia M (2019). Extending Janzen’s hypothesis to temperate regions: a test using subterranean ecosystems. Funct. Ecol..

[69] Kratochvíl J (1978). Araignées cavernicoles des îles Dalmates. Přírodovědné práce ústavů Československé Akad. Věd v Brně.

[70] Denny M (2017). The fallacy of the average: on the ubiquity, utility and continuing novelty of Jensen’s inequality. J. Exp. Biol..

[71] Mammola S (2019). Figshare.

[72] Darwin, C. *On the origin of species by means of natural selection, or the preservation of favoured races in the struggle of life*. (John Murray, 1859). PMC5184128 30164232

[73] Wong MKL, Guénard B, Lewis OT (2019). Trait-based ecology of terrestrial arthropods. Biol. Rev..

[74] Lučić I (2021). Interview with Boris Sket: nothing has a sense in speleobiology, without a comparison of cave animals with the ‘normal’ epigean ones. Acta Carsologica.

[75] McGill BJ (2019). The what, how and why of doing macroecology. Glob. Ecol. Biogeogr..

[76] Muscarella R, Uriarte M (2016). Do community-weighted mean functional traits reflect optimal strategies?. Proc. R. Soc. B Biol. Sci..

[77] Petchey OL, Gaston KJ (2002). Functional diversity (FD), species richness and community composition. Ecol. Lett..

[78] Mammola S, Cardoso P (2020). Functional diversity metrics using kernel density n-dimensional hypervolumes. Methods Ecol. Evol..

[79] Mammola S (2019). Local- versus broad-scale environmental drivers of continental β-diversity patterns in subterranean spider communities across Europe. Proc. R. Soc. B Biol. Sci..

[80] Graco-Roza, C. *et al*. Distance decay 2.0 – a global synthesis of taxonomic and functional turnover in ecological communities. *Glob. Ecol. Biogeogr*, in press (available at 10.1101/2021.03.17.435827) (2022). 10.1111/geb.13513 PMC9322010 35915625

[81] Gallagher RV (2021). A guide to using species trait data in conservation. One Earth.

[82] Chichorro F, Juslén A, Cardoso P (2019). A review of the relation between species traits and extinction risk. Biol. Conserv..

[83] Chichorro, F. *et al*. Species traits predict extinction risk across the Tree of Life. *bioRxiv* 2020.07.01.183053 (2020).

[84] Violle C (2017). Functional rarity: the ecology of outliers. Trends Ecol. Evol..

[85] Carmona CP (2021). Erosion of global functional diversity across the tree of life. Sci. Adv..

[86] Loreau M (2021). Biodiversity as insurance: from concept to measurement and application. Biol. Rev..

[87] Sánchez-Fernández D, Galassi DMP, Wynne JJ, Cardoso P, Mammola S (2021). Don’t forget subterranean ecosystems in climate change agendas. Nat. Clim. Chang..

[88] Borges PAV (2012). Volcanic caves: Priorities for conserving the Azorean endemic troglobiont species. Int. J. Speleol..

[89] Rabelo LM, Souza-Silva M, Ferreira RL (2018). Priority caves for biodiversity conservation in a key karst area of Brazil: comparing the applicability of cave conservation indices. Biodivers. Conserv..

[90] Nitzu E (2018). Assessing preservation priorities of caves and karst areas using the frequency of endemic cave-dwelling species. Int. J. Speleol..

[91] Pipan T, Deharveng L, Culver DC (2020). Hotspots of subterranean biodiversity. Diversity.

[92] Fattorini S, Fiasca B, Di Lorenzo T, Di Cicco M, Galassi DMP (2020). A new protocol for assessing the conservation priority of groundwater-dependent ecosystems. Aquat. Conserv. Mar. Freshw. Ecosyst..

[93] Iannella, M. *et al*. Getting the ‘most out of the hotspot’ for practical conservation of groundwater biodiversity. *Glob. Ecol. Conserv*. e01844 (2021).

[94] Mazel F (2018). Prioritizing phylogenetic diversity captures functional diversity unreliably. Nat. Commun..

[95] Cadotte MW, Tucker CM (2018). Difficult decisions: Strategies for conservation prioritization when taxonomic, phylogenetic and functional diversity are not spatially congruent. Biol. Conserv..

[96] Hanson JO (2020). Global conservation of species’ niches. Nature.

[97] Pollock LJ (2020). Protecting biodiversity (in all its complexity): new models and methods. Trends Ecol. Evol..

[98] Mammola S (2019). Scientists’ warning on the conservation of subterranean ecosystems. Bioscience.

[99] Wynne JJ (2021). A conservation roadmap for the subterranean biome. Conserv. Lett..

[100] Mammola, S. *et al*. Towards evidence-based conservation of subterranean ecosystems. *Biol. Rev*., early view at 10.1111/brv.12851 (2022). 10.1111/brv.12851 PMC9545027 35315207

[101] Culver, D. C. & Pipan, T. *The biology of caves and other subterranean habitats*. (Oxford University Press, USA, 2014).

[102] Culver, D. C. & Pipan, T. *Shallow Subterranean Habitats: Ecology, Evolution, and Convervation*. (Oxford University Press, USA, 2014).

[103] Sobral M (2021). All traits are functional: an evolutionary viewpoint. Trends Plant Sci..

[104] Pipan T, Culver DC (2017). The unity and diversity of the subterranean realm with respect to invertebrate body size. J. Cave Karst Stud..

[105] Elgar MA, Ghaffar N, Read AF (1990). Sexual dimorphism in leg length among orb-weaving spiders: a possible role for sexual cannibalism. J. Zool..

[106] Deeleman-Reinhold, C. L. Revision of the cave-dwelling and related spiders of the genus *Troglohyphantes* Joseph (Linyphiidae), with special reference to the Yugoslav species. *Opera Acad. Sci. Artium Slov*. **23** (1978).

[107] Isaia M, Pantini P (2010). New data on the spider genus *Troglohyphantes* (Araneae, Linyphiidae) in the Italian Alps, with the description of a new species and a new synonymy. Zootaxa.

[108] Hagstrum DW (1971). Carapace width as a tool for evaluating the rate of development of spiders in the laboratory and the field. Ann. Entomol. Soc. Am..

[109] Pavlek M, Mammola S (2020). Niche-based processes explaining the distributions of closely related subterranean spiders. J. Biogeogr..

[110] Mammola S (2017). Modelling the future spread of native and alien congeneric species in subterranean habitats - The case of meta cave-dwelling spiders in Great Britain. Int. J. Speleol..

[111] Novak T (2010). Niche partitioning in orbweaving spiders *Meta menardi* and *Metellina merianae* (Tetragnathidae). Acta Oecologica.

[112] Lunghi E (2020). Occurrence of the Black lace-weaver spider, Amaurobius ferox, in caves. Acta Carsologica.

[113] Isaia M, Chiarle A (2015). Taxonomic notes on *Cybaeus vignai* Brignoli, 1977 (Araneae, Cybaeidae) and *Dysdera cribrata* Simon, 1882 (Araneae, Dysderidae) from the Italian Maritime Alps. Zoosystema.

[114] Ledford J (2021). Phylogenomics and biogeography of leptonetid spiders (Araneae: Leptonetidae). Invertebr. Syst..

[115] Isaia M, Mammola S, Mazzuca P, Arnedo MA, Pantini P (2017). Advances in the systematics of the spider genus *Troglohyphantes* (Araneae, Linyphiidae). Syst. Biodivers..

[116] Hajer J, Řeháková D (2003). Spinning activity of the spider Trogloneta granulum (Araneae, Mysmenidae): web, cocoon, cocoon handling behaviour, draglines and attachment discs. Zoology.

[117] Huber BA, Pavlek M, Komnenov M (2021). Revision of the spider genus Stygopholcus (Araneae, Pholcidae), endemic to the Balkan Peninsula. Eur. J. Taxon..

[118] Huber BA (2020). Revision of the spider genus *Hoplopholcus* Kulczyński (Araneae, Pholcidae). Zootaxa.

[119] Cardoso P, Scharff N (2009). First record of the spider family symphytognathidae in Europe and description of *Anapistula ataecina* sp. n. (araneae). Zootaxa.

[120] Wang C, Ribera C, Li S (2012). On the identity of the type species of the genus *Telema* (Araneae, Telemidae). Zookeys.

[121] Hesselberg, T., Simonsen, D. & Juan, C. Do cave orb spiders show unique behavioural adaptations to subterranean life? A review of the evidence. *Behaviour* 1–28 (2019).

